# Syntenin Regulated by miR-216b Promotes Cancer Progression in Pancreatic Cancer

**DOI:** 10.3389/fonc.2022.790788

**Published:** 2022-01-28

**Authors:** Fuqiang Zu, Hui Chen, Qingfeng Liu, Hui Zang, Zeyu Li, Xiaodong Tan

**Affiliations:** ^1^ Department of Pancreatic and Thyroid Surgery, General Surgery, Shengjing Hospital of China Medical University, Shenyang, China; ^2^ Department of General Surgery, The People’s Hospital of China Medical University, Shenyang, China

**Keywords:** syntenin, miR-216b, progression, EMT, pancreatic cancer

## Abstract

Outcomes for patients with pancreatic cancer (PC) are poor; therefore, there is an urgent need to identify novel therapeutic targets involved in the progression of PC. We previously identified 161 differentially expressed proteins (DEPs) in PC. Syntenin (SDCBP) was identified as a survival-related protein through integrated, survival, and Cox analyses. High expression of SDCBP was associated with a poor prognosis in PC tissue and promoted the proliferation, migration, and invasion of PC cells, and induced epithelial–mesenchymal transition (EMT) *via* the PI3K/AKT pathway. Additionally, we elucidated the regulatory mechanism underlying these roles of SDCBP at the post-transcriptional level. microRNAs (miRNAs) of SDCBP were predicted using bioinformatics. Low levels of miR-216b expression were confirmed in PC tissues and were negatively correlated with SDCBP expression. miR-216b was found to directly regulate SDCBP expression through luciferase reporter assays. Furthermore, agomiR-216b restrained PC proliferation, migration, invasion, and EMT *via* the PI3K/AKT pathway, whereas antagomiR-216b facilitated this process. Notably, the knockout of SDCBP counteracted the effect of antagomiR-216b in PC, which suggested that miR-216b and SDCBP represent molecular targets underlying PC progression and EMT. Finally, the results were validated in *in vivo* studies. These findings indicated that low expression of miR-216b and the oncogene SDCBP contributes to PC migration, invasion, and EMT, and that they have potential as future therapeutic targets for patients with PC.

## Introduction

Pancreatic cancer (PC) is associated with a high mortality rate, high recurrence rate, and low cure rate, ranking eleventh in morbidity and sixth in mortality among all cancers (1, 2). Based on GLOBOCAN 2018 estimates, there were approximately 459,000 new cases of PC and 432,000 deaths each year (3). The high mortality and recurrence of PC make the disease difficult to treat. Furthermore, approximately 80%–85% of patients are diagnosed with unresectable or metastatic disease, which is associated with poor overall survival (OS) (4). Despite improvements in OS due to advances in diagnostic approaches, surgical techniques, and adjuvant therapy, outcomes for patients with PC remain unsatisfactory, with only 10% surviving 5 years (5, 6). Therefore, there is an urgent need to identify novel therapeutic targets involved in the progression of PC.

The invasive and metastatic potential of homologous PC cell lines differs; for example, PC-1.0 cells present high invasive and metastatic potential, while PC-1 cells present lower potential. Previously, we identified 161 differentially expressed proteins (DEPs) between PC-1.0 and PC-1 cell lines through proteomic analysis (7). In that study, we elucidated the roles of these DEPs in PC, and evaluated their expression and relationship with prognosis through bioinformatic analyses. Overall, three proteins were validated, namely, CLIC1, KRT7, and syntenin (SDCBP). CLIC1 and KRT7 are involved in PC migration and invasion (8, 9); however, the role of SDCBP in PC remains unclear. Syntenin is a multifunctional scaffold protein, which contains two PDZ domains and promotes cellular migration and invasion through epithelial–mesenchymal transition (EMT) (10–12). During EMT, epithelial cells are transformed to a mesenchymal phenotype, which endows them with the ability to metastasize and invade (13, 14). This process is a key step in the progression and metastasis of PC (15–17). Furthermore, SDCBP exerts a regulatory effect on EMT biomarkers, such as E-cadherin, N-cadherin, and ZO-1 (18–20); however, the relationship between SDCBP and EMT in PC remains unclear.

MicroRNAs are highly conserved short-chain non-coding RNAs about 21–25 nucleotides in length, and are powerful regulators of tumor development, progression, and metastasis (21–23). Evidence suggests that microRNAs act as biomarkers in PC and exert crucial regulatory roles in disease progression (24–26). Among them, miR-216b functions in multiple cancer types, including PC (27–29). For example, low expression of miR-216b is associated with large tumor size, advanced tumor node metastasis (TNM) stage, and poor OS in patients with PC (30). MiR-216b was found to inhibit PC progression and promote apoptosis by suppressing KRAS expression (31). However, the relationship between miR-216b and SDCBP in PC remains to be elucidated. Therefore, we evaluated the expression of SDCBP in PC tissues and confirmed its stimulatory role in the proliferation, migration, and invasion, and in the induction of EMT through the PI3K/AKT pathway. Additionally, miR-216b was found to target SDCBP and regulate its carcinogenic effect in PC tissues. Notably, knockout of SDCBP was able to counteract the effects of antagomiR-216b in PC, highlighting novel molecular mechanisms involved in the migration and invasion of PC. Our findings indicated that the miR-216b/SDCBP axis promotes PC progression and induced EMT *via* the PI3K/AKT pathway, which may represent potential future therapeutic targets.

## Materials and Methods

### Identification of Survival-Related Proteins

Previously, we identified 161 DEPs with a ≥1.5-fold change (FC) in PC *via* proteomic analyses (7). The effect of DEP expression on the prognosis of patients with PC was evaluated using TCGA, GTEx, and GEO databases (32–34). Due to the limited number of normal samples in TCGA, TCGA and GTEx were combined to form the TCGA_GTEx group. Raw RNA-seq data for the TCGA_GTEx group were acquired from the UCSC database, and included 178 PC samples and 171 pancreas samples (35). To reduce bias associated with the use of a single database, two GEO datasets were selected for further analysis: GSE62165 and GSE15471. Stepwise, all data were log2 (*x* + 1) transformed and normalized *via* the “LIMMA” package (36). Differentially expressed genes (DEGs) were obtained using the “LIMMA” package with thresholds of |log2 FC| > 1, with adjusted *p* < 0.05. Differentially expressed miRNAs (DEMIs) were screened using the “edgR” package with the criteria of |log2FC| > 1 and adjusted *p* < 0.05 (37). Additionally, the Kaplan–Meier analysis was applied to evaluate OS. Univariate and multivariate Cox proportional hazards regression (CPHR) analyses were used to evaluate the impact on survival. Only common protein expression was consistent with prognosis and a *p*-value lower than 0.05 was regarded as survival-related proteins. Finally, SDCBP was selected for subsequent analysis.

### Functional Analysis and miRNA Prediction

Gene set enrichment analysis (GSEA) was performed to determine SDCBP function in PC. The potential function of SDCBP was determined through Gene Ontology (GO) term analysis and Kyoto encyclopedia of genes and genomes (KEGG) pathway analysis with a cutoff criterion of *p* < 0.05 (38, 39). Next, the top five most enriched GO terms and KEGG pathways were identified. In addition, upstream miRNAs of SDCBP were predicted by TargetScan and miRNet databases (40, 41). To improve reliability, miRNAs with low expression in PC were selected for further analysis. Additionally, we evaluated OS through Kaplan–Meier analysis. Finally, correlation analysis was applied to calculate the correlation index of SDCBP with miRNAs with a cutoff criterion of *p* < 0.05. The qualified miRNAs were selected for further analysis.

### Tissue Samples and Cell Lines

To detect the prognostic ability of SDCBP in PC, we collected 61 paraffin-embedded samples for which complete clinical data were available from Shengjing Hospital of China Medical University between 2013 and 2016. All samples were obtained from patients who underwent radical surgery for PC, with diagnosis confirmed by a pathologist. Patients with complete clinicopathological data and follow-up data were included. Additionally, to determine the expression of SDCBP through Western blot (WB) and quantitative polymerase chain reaction (qPCR) assays, a further 12 pairs of fresh PC and normal tissues were obtained between 2019 and 2020. All specimens were collected following surgical resection and immediately frozen at −80°C until use. All patients signed written informed consent before enrollment, and the project was approved by the Ethics Committee of Shengjing Hospital of China Medical University (2020PS617K).

Cell lines were purchased from the National Collection of Authenticated Cell cultures (Shanghai, China) and included Aspc-1, BxPC-3, Capan-2, PANC-1, and SW1990 cell lines, and the pancreatic cell line HPDE. The HPDE cell line was established from ductal epithelial cells of the pancreas. The Aspc-1 cell line was established using metastatic cells from PC ascites carrying CDKN2A, FBXW7, KRAS, MAP2K4, and TP53 mutations. The BxPC-3 cell line was established from primary PC carrying mutations of CDKN2A, MAP2K4, SMAD4, and TP53. The Capan-2 cell line was established from primary PC carrying KRAS mutation. The PANC-1 cell line was established from primary PC carrying a KRAS and TP53 mutation. The SW1990 cell line was established from PC using spleen metastatic cells containing CDKN2A and KRAS mutations (42, 43). Aspc-1, BxPC-3, Capan-2, and SW1990 cells were cultured in RPIM1640 medium containing 10% fetal bovine serum (FBS, Gibco, USA), whereas PANC-1 and HPDE cells were maintained in DMEM with 10% FBS. All cells were cultured at 37°C in a humidified incubator supplemented with 5% CO_2_.

### Immunohistochemistry

SDCBP expression in tissues was determined using a two-step immunohistochemistry kit (E-IR-R217, Elabscience, USA), according to the manufacturer’s instructions. Briefly, tissue slices were dewaxed with xylene and hydrated through graded ethanol. Then, slices were heated to retrieve antigens, covered with 3% hydrogen peroxide to inactivate endogenous enzymes, and blocked with normal goat serum. After this, slices were incubated overnight at 4°C with anti-SDCBP (1:200, Proteintech, USA) and incubated with polyperoxidase anti-Rabbit IgG at 37°C for 30 min. Finally, 3,3-diaminobenzidine reagent was applied during the color development stage to visualize staining, and slices were sealed with neutral balsam. Staining intensity was graded as 0 (negative), 1 (weak), 2 (medium), or 3 (strong). Staining extent was scored according to the percentage of positive cells as follows: 0 (<5%), 1 (5%–25%), 2 (26%–50%), 3 (51%–75%), and 4 (>75%). Three fields were randomly selected and used to calculate the average score. The intensity and extent scores were multiplied as the final staining scores (0–12). A final score of >2 was considered as high expression, while others were considered as low expression.

### Western Blot Analysis

For WB analysis, samples were lysed in RIPA lysis buffer (Solarbio, China) containing 1% protease inhibitor (Solarbio, China) and 1% phosphatase inhibitor (Solarbio, China), and centrifuged at 14,000 rpm for 30 min at 4°C. Protein concentration was determined using the BCA protein assay Kit (Solarbio, China). Then, 30 μg of total protein was loaded subjected to SDS-PAGE (10%) and transferred to a polyvinylidene difluoride membrane (Millipore, USA). The membranes were blocked with 5% bovine serum albumin (BSA) for 2 h at room temperature, incubated with primary antibodies overnight at 4°C, and incubated with secondary antibodies for 2 h at room temperature. Finally, protein bands were visualized using an ECL detection kit under a luminescent image analyzer. The following antibodies were used: anti-SDCBP (1:1,000, Proteintech, USA), anti p-PI3K (1:1,000, Affinity, USA), anti-PI3K (1:1,000, Affinity, USA), anti p-AKT (1:2,000, Affinity, USA), anti-AKT (1:5,000, Proteintech, USA), anti E-cadherin (1:5,000, Proteintech, USA), anti N-cadherin (1:5,000, Proteintech, USA), anti-GAPDH (1:10,000, Proteintech, USA), and anti-β-actin (1:10,000, Proteintech, USA).

### Quantitative Real-Time PCR

Total RNA was extracted with RNAiso Plus reagent (Takara, Japan) and dissolved in 10 μl of RNase-free water. The concentration and purity of total RNA were measured using a NanoPhotometer 50 (Implen, Germany). For mRNA reverse transcription, 1.0 μg of the total RNA was subjected to the HiScript III RT SuperMix (Vazyme, China). For miRNA reverse transcription, the miRNA 1st Strand cDNA Synthesis Kit (Vazyme, China) was used. The reaction conditions were as follows: 37°C for 15 min, 85°C for 5 s, followed by cooling to 4°C. Then, cDNA was subjected to qPCR on an ABI 7500 RT-PCR system (Thermo Fisher, USA). The amplification system consisted of 2 μl of cDNA, 0.4 μl each of forward and reverse primer, 10 μl of ChamQ Universal SYBR qPCR Master Mix for mRNA or miRNA Universal SYBR qPCR Master Mix for miRNA, and ddH_2_O up to a final volume of 20 μl. The amplification conditions involved mRNA denaturation at 95°C for 30 s or miRNA denaturation at 95°C for 5 min, followed by 40 cycles of 95°C for 10 s and 60°C for 30 s. Primer specificity was determined by melting curve analysis. GAPDH and β-actin were used as endogenous reference genes for mRNA in tissue and cell PCR, respectively, and U6 was used as an endogenous reference for miRNA. Relative expression levels were quantified using the 2^−ΔΔ^Ct method. The primers were synthesized by Sangon (China), and the sequences are as follows: SDCBP: 5’-CTG CTC CTA TCC CTC ACG ATG-3’ (Forward), 5’-GGC CAC ATT TGC ACG TAT TTC T-3’ (Reverse). GAPDH: 5’-GTC TCC TCT GAC TTC AAC AGC G-3’ (Forward), 5’-ACC ACC CTG TTG CTG TAG CCA A-3’ (Reverse). β-actin: 5’-CAC CAT TGG CAA TGA GCG GTT C-3’ (Forward), 5’-AGG TCT TTG CGG ATG TCC ACG T-3’ (Reverse). hsa-miR-216b: 5’-AAA UCU CUG CAG GCA AAU GUG A-3’ (Forward), 5’-TTT AGA GAC GTC CGT TTA CAC T-3’ (Reverse). RT Primer: 5’-GTC GTA TCC AGT GCA GGG TCC GAG GTA TTC GCA CTG GAT ACG ACT CAC AT-3’. U6: 5’-CGC AAG GAT GAC ACG CAA AT-3’ (Forward), 5’-CGG CAA TTG CAC TGG ATA CG-3’ (Reverse).

### Cell Transfection and Luciferase Reporter

SDCBP-shRNA lentiviral vectors (hU6-MCS-CBh-gcGFP-IRES-puromycin) were purchased from GeneChem (Shanghai, China). Capan2 and SW1990 cells were transfected according to the manufacturer’s instructions. The cells were subjected to puromycin selection for 1 week, after which the surviving cells were used for further experiments. For miRNA transfection, the negative control (NC), agomir, and antagomir of hsa-miR-216b (agomiR-216b and antagomiR-216b) were synthesized from GenePharma (Shanghai, China), and transfected into Capan2 and SW1990 cells using Lipofectamine 3000 (Invitrogen, USA). For the luciferase assay, we identified hsa-miR-216b as a direct upstream miRNA in SDCBP and predicted its binding sites using the TargetScan database. The wild-type 3ʹ-untranslated region (UTR) of SDCBP and mutant 3ʹ-UTR SDCBP were constructed by GenePharma and cloned into the pmir-GLO-luciferase reporter plasmid. Capan2 and SW1990 were co-transfected with wild-type and mutant pmir-GLO-SDCBP, and miR-216b mimics or NC using Lipofectamine 3000. After 48 h, cell lysis buffer was collected, and luciferase activity was determined using the Dual-Luciferase Reporter Gene Assay Kit (GenePharma, China). Luciferase activity was normalized to that of Renilla.

### Cell Proliferation Assay

The cell counting kit-8 (CCK-8) assay is used widely to evaluate cell proliferation and cytotoxicity (44, 45). Cell proliferation was evaluated by the CCK-8 assay according to instructions (Solarbio, China). Briefly, cells were transfected, plated, and grown in 96-well plates. Then, cell viability was determined at 0, 24, 48, 72, and 96 h using CCK-8. The absorbance was determined at 450 nm using a microplate reader (BioTek, USA). All samples were run in triplicate.

### Colony Formation Assay

For the colony formation assay, 1,000 cells were seeded in six-well plates and then cultured for 1 week. Next, colonies were fixed with 4% paraformaldehyde for 1 h and stained with 1% crystal violet for 1 min. Only clones containing more than 50 cells were counted and analyzed.

### Wound-Healing and Transwell Assays

Cell migration and invasion were evaluated through wound-healing and Transwell invasion assays, respectively. To assess wound healing, cells were plated on six-well plates and cultured until they reached 90%–100% confluency. Then, the cells were scraped using a sterile 200-μl pipette tip. Scratched areas were photographed after 0 and 24 h at 100× magnification using an inverted microscope (Nikon DS- Ri2, Japan). For the Transwell invasion assay, 800 μl of medium containing 10% FBS was added to the lower chamber of a Transwell plate as a chemoattractant, and 200 μl of serum-free medium was added to the upper chamber, which was coated with Matrigel (Corning, USA). Then, cells were seeded onto the upper chamber and plates were incubated for 24 h.

Cells traversing the membranes were fixed and stained with a Hematoxylin-Eosin Staining Kit (Solarbio, China), according to the manufacturer’s instructions. Images of cells were captured using a microscope (Nikon E800) at 200× magnification, and five random fields were counted and analyzed.

### Tumorigenesis Experiment

The protocol for the animal experiment was approved by the Ethics Committee at Shengjing Hospital of China Medical University (2020PS617K). Sixteen female BALB/c nude mice (8 weeks old, 18-20 g) were purchased from HFk Bioscience (Beijing, China), and randomly divided into four groups, each containing four mice. Animals were housed in a specific pathogen-free facility under suitable growth conditions. We evaluated the roles of SDCBP and miR-216b in tumor growth *via* a subcutaneous experiment in nude mice. To generate xenograft tumors *in vivo*, 1 × 10^6^ cells of each group were subcutaneously injected in the left axillary of mice. Tumor volume (V) was measured at 7, 14, and 21 days according to the following equation: V = π/6 × L × W^2^, where L and W denote the long and short axis of the tumor, respectively. Finally, all mice were euthanized and dissected. Tumors were weighed and images were obtained for further analyses.

### Statistical Analysis

All statistical analyses were performed using GraphPad Prism 8.0 (IBM, USA) and the R Software (version 3.6.1). Data are expressed as the mean ± standard deviation (SD). Significant differences were analyzed using Student’s *t*-test to compare two groups, and one-way ANOVA to compare multiple groups. Survival curves were prepared using the Kaplan–Meier analysis and analyzed using the log-rank test. Correlations between miRNA and SDCBP were evaluated through Pearson correlation analysis. All experiments were repeated three times. *p* < 0.05 was considered to be statistically significant (*p < 0.05).

## Results

### Identification of Three Survival-Related Proteins

Details of 161 DEPs are shown in **Supplementary Table S1**. A total of 332 PC samples and 220 normal samples were used to validate the results. Details of each group are presented in **Supplementary Table S2**. Briefly, there were 5,624 DEGs in the TCGA_GTEx group, 3,117 DEGs in the GSE62165 group, and 1,492 DEGs in the GSE15471 group based on |log2FC| >1 and adjusted *p* < 0.05, with 21 common DEGs among the four groups (**Supplementary Figure S1** and **Supplementary Table S1**). We evaluated the expression and prognostic effect of 21 DEPs through Kaplan–Meier analysis, univariate CPHR, and multivariate CPHR. Eight DEPs were found to be upregulated in PC and were associated with poor survival. After univariate and multivariate CPHR, only three DEPs (CLIC1, KRT7, and SDCBP) were validated as survival-related proteins in PC (**Supplementary Figure S2**). Due to limited research on SDCBP in PC, this was subsequently selected for further analysis.

### GSEA Analysis and miRNA Prediction

GSEA was performed to determine the functional roles of SDCBP in PC. The top five GO terms and KEGG pathways are shown in **Supplementary Figure S2**. Briefly, the GO terms indicated that SDCBP was most enriched in cell adhesion, extracellular matrix binding, and regulation of epithelial cell migration (**Supplementary Figure S2**). KEGG pathway analysis revealed that SDCBP was detected in the PI3K-AKT signaling, Jak-STAT signaling, and ECM-receptor interaction pathways (**Supplementary Figure S2G**). These predictions suggested that SDCBP might be involved in the PI3K-AKT signaling pathway and in EMT. Additionally, upstream miRNAs of SDCBP were predicted through the interaction of TargetScan database, miRNet database, and differential expression miRNAs (DEMIs) with thresholds of |log2 FC| > 1 and adjusted *p* < 0.05 in TCGA database. Overall, we found 279 target miRNAs of SDCBP in TargetScan, 70 target miRNAs in miRNet, and 37 DEMIs (**Supplementary Figures S3A, B**). Three common target miRNAs were identified, including hsa-miR-139, hsa-miR-155, and hsa-miR-216b. Next, we evaluated the expression and prognostic value of target miRNAs. Only miR-216b was found to have low expression and was related to poor survival in PC (**Supplementary Figures S3C, D**). Finally, we validated the inter-relationships between SDCBP and target miRNAs through correlation analysis. Our results indicated that SDCBP was most strongly correlated with miR-216b expression with a relation index (R) equal to −0.34 and *p* < 0.05 (**Supplementary Figure S3E**). Therefore, we identified miR-216b as the upstream miRNAs of SDCBP in PC, and this was selected for further analysis.

### SDCBP Expression Indicated a Dismal Survival

**Figure 1:**
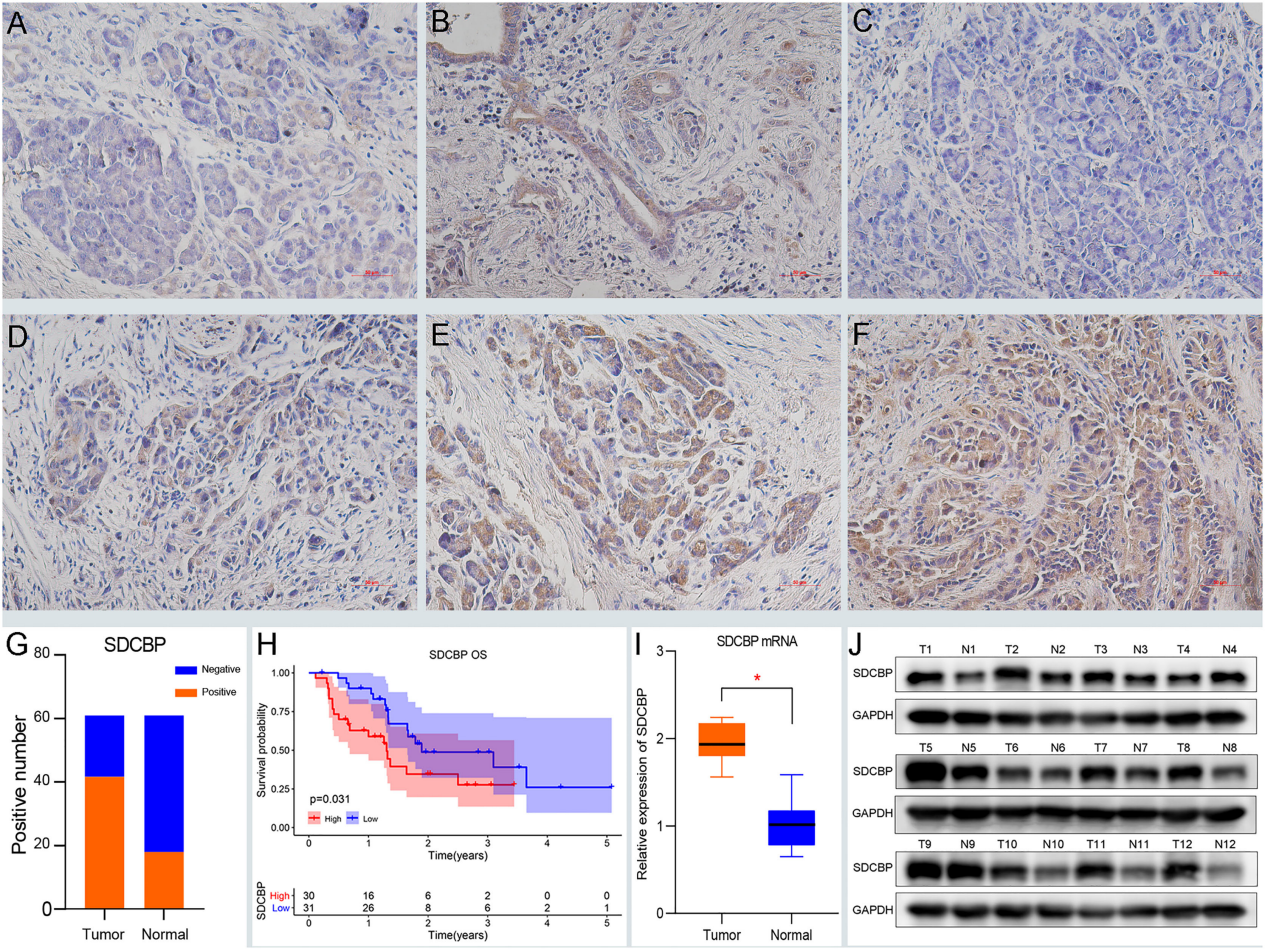
High expression of SDCBP indicates a dismal survival. **(A, B)** The negative **(A)** and positive **(B)** expression of SDCBP in pancreatic tissues, respectively. **(C–F)** SDCBP expression in pancreatic cancer (PC) tissues including negative expression **(C)**, weak expression **(D)**, moderate expression **(D)**, and strong expression **(F)**, respectively (×200 magnification). **(G)** The positive rates of SDCBP in PC compared to pancreatic tissues. **(H)** The overall survival of SDCBP in our cohort. **(I, J)** The expression of SDCBP in PC and pancreatic tissues using qRT-PCR **(I)** and WB assay **(J)**. *Means P < 0.05.

Based on previous predictions, we determined the expression and prognostic roles of SDCBP *via* immunohistochemistry. Samples were divided into two groups: SDCBP high expression and SDCBP low expression. The clinical data are shown in **Supplementary Table S3**. IHC revealed high expression of SDCBP in 67.2% (41/61) of PC tissues, compared with 29.5% (18/61) of normal pancreatic tissues (**Figure 1G**). In normal pancreas tissue, SDCBP was mainly expressed in the acinar cells, with some expression observed in epithelial ductal cells (**Figures 1A, B**). In PC tissues, SDCBP expression was mainly located in the membrane and cytoplasm (**Figures 1C–F**). Additionally, high expression of SDCBP was associated with poor survival with *p* = 0.031 (**Figure 1H**). WB and RT-qPCR analyses revealed that SDCBP was more highly expressed in PC tissues compared with normal tissues (**Figures 1I, J**).

### SDCBP Promoted PC Progression *via* the PI3K/AKT Pathway

**Figure 2:**
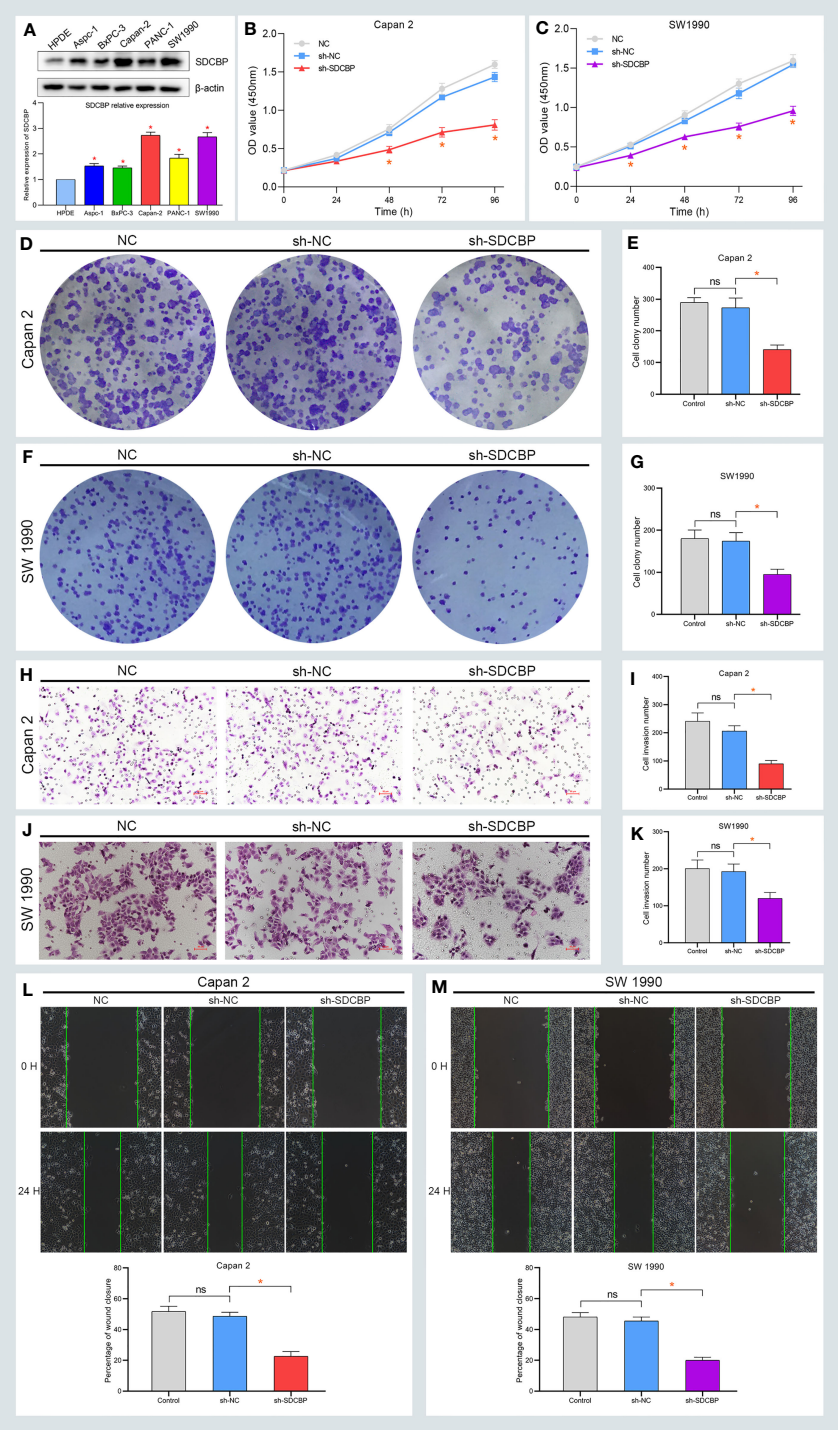
High expression of SDCBP promotes PC proliferation, migration, and invasion. **(A)** The relative expression of SDCBP was evaluated in HPDE cells and 5 PC cells. **(B, C)** SDCBP knockdown inhibited the proliferation of Capan-2 cell **(B)** and SW1990 cell **(C)** through CCK-8 assay, respectively. **(D–G)** The number of colony formation in Capan-2 cell **(D, E)** and SW1990 cell **(F, G)** was separately repressed after SDCBP knockdown. **(H–K)** SDCBP inhibition restrained cell invasion ability in both Capan-2 cell **(H, I)** and SW1990 cell **(J, K)**, respectively. **(L, M)** The migration ability of PC cells was inhibited after SDCBP knockdown. ns means no statistically significant. *Means P < 0.05.

**Figure 3:**
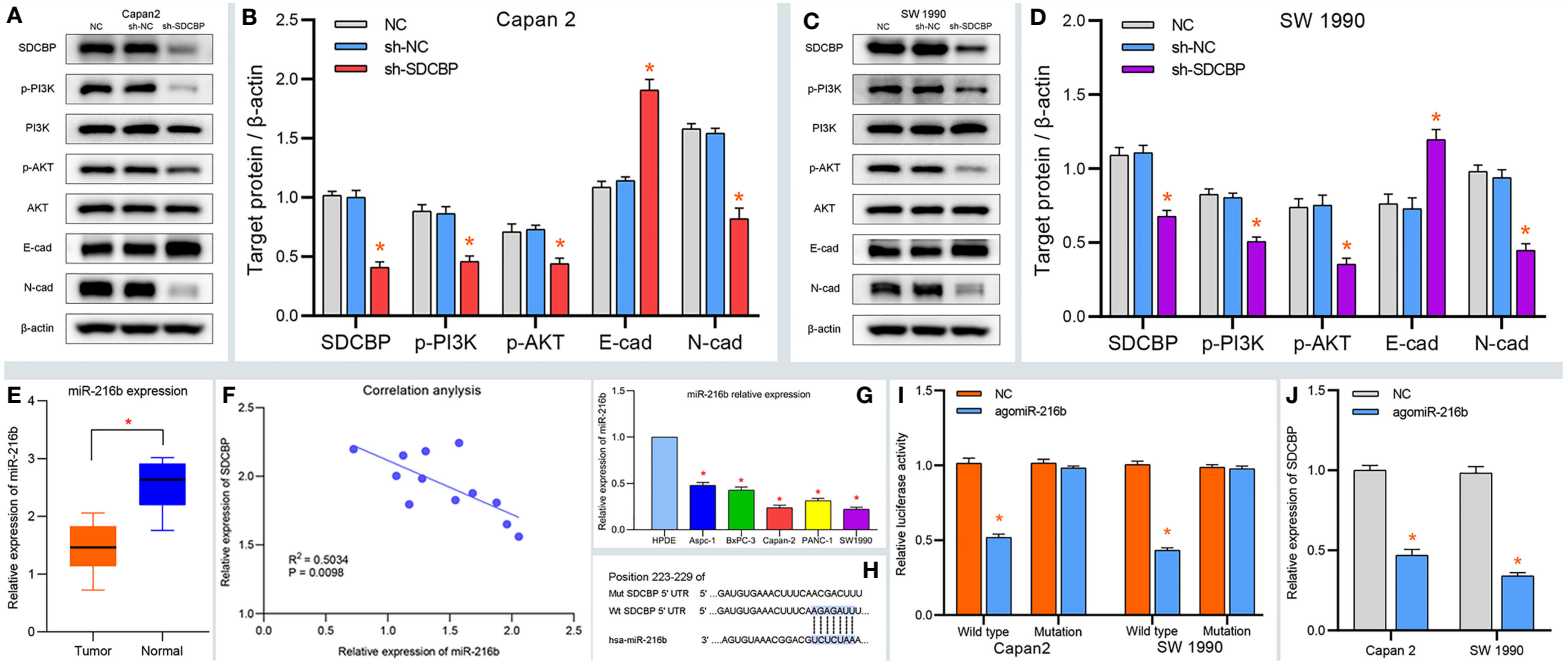
SDCBP induces PC EMT *via* PI3K/AKT pathway and miRNA validation. **(A–D)** The protein levels of p-PI3K, p-AKT, and N-cadherin (N-cad) were downregulated after SDCBP knockdown, whereas E-cadherin protein levels were upregulated, and there were no changes in PI3K and AKT protein levels. **(E)** The relative expression of miR-216b in PC tissues compared with pancreatic tissues. **(F)** The correction between SDCBP and miR-216b in PC tissues. **(G)** The relative expression of miR-216b in HPC-Y5 cell and PC cells. **(H)** The binding sites of miR-216b with wild type and mutant SDCBP. **(I)** The luciferase activity of wild-type SDCBP reduced after agomiR-216b co-transfection. **(J)** The mRNA levels of SDCBP changed in agomiR-216b transfection. *Means P < 0.05.

To explore the role of SDCBP in PC, we evaluated the expression of SDCBP in one pancreatic cell line HPDE, and five PC cell lines using WB and RT-qPCR analyses. As shown in **Figure 2A**, high levels of SDCBP expression were observed in Capan-2 and SW1990 cells. Next, we knocked down SDCBP expression using lentivirus and evaluated its roles in PC progression and the underlying mechanism of action. Cell proliferation, migration, and invasion were evaluated after SDCBP knockdown. As shown in **Figures 2B, C**, the proliferation of Capan-2 and SW1990 cells was markedly inhibited after SDCBP knockdown, which indicated that SDCBP could promote PC proliferation. For the colony formation assay, SDCBP knockdown repressed PC proliferation and colony formation in both Capan-2 and SW1990 cells (**Figures 2D, F**); this repression was statistically significant (*p* < 0.05; **Figures 2E, G**). The results of the Transwell invasion assay suggested that SDCBP inhibition significantly attenuated the invasion ability of the cells (**Figures 2H–K**). Moreover, the migratory ability of PC cells was also inhibited following SDCBP knockdown (**Figures 2L, M**). However, it is unknown why SDCBP knockdown inhibited PC proliferation, migration, and invasion. Based on the results of our studies, we investigated the relationship among SDCBP expression, EMT, and the PI3K/AKT pathway. As shown in **Figures 3A–D**, the expression of a number of proteins was markedly downregulated following SDCBP knockdown, including that of p-PI3K, p-AKT, and N-cadherin. However, no changes in the expression of PI3K and AKT proteins were observed. Notably, the protein expression of E-cadherin was markedly upregulated, while that of N-cad was inhibited, which represent important features of mesenchymal transformation (EMT). Thus, SDCBP might promote PC progression *via* EMT mediated by the PI3K/AKT pathway.

### MiR-216b Identified as a Target miRNA of SDCBP

MicroRNAs are important regulators of protein expression and play essential roles in the progression of PC. To elucidate the relationship between SDCBP and miRNAs, we predicted and validated the upstream miRNA of SDCBP. As shown in **Supplementary Figures S3B–E**, miR-216b was predicted as a target miRNA of SDCBP. To validate our prediction, the relative expression of miR-216b was determined in PC tissues. The results suggested that miR-216b was expressed at low levels in PC tissues (**Figure 3E**). Correlation analysis revealed that miR-216b was negatively correlated with SDCBP expression (**Figure 3F**), which was consistent with our prediction (**Supplementary Figure S3E**). In addition, low expression of miR-216b was also found in Capan-2 and SW1990 cells (**Figure 3G**). Next, the binding sites of miR-216b and SDCBP were predicted (**Figure 3H**) and a luciferase plasmid was constructed with wild-type and mutant SDCBP. The luciferase activity of wild-type SDCBP was notably reduced following co-transfection with agomiR-216b in Capan-2 and SW1990 cells. However, the luciferase activity of mutant SDCBP was unchanged (**Figure 3I**). Thus, miR-216b could directly regulate SDCBP expression. Notably, the protein and RNA expression of SDCBP was reduced following transfection with agomiR-216b (**Figures 3J** and **5A, C**). Overall, these findings revealed that miR-216b might regulate PC progression by directly targeting SDCBP.

### MiR-216b Regulated PC Progression *via* SDCBP

**Figure 4:**
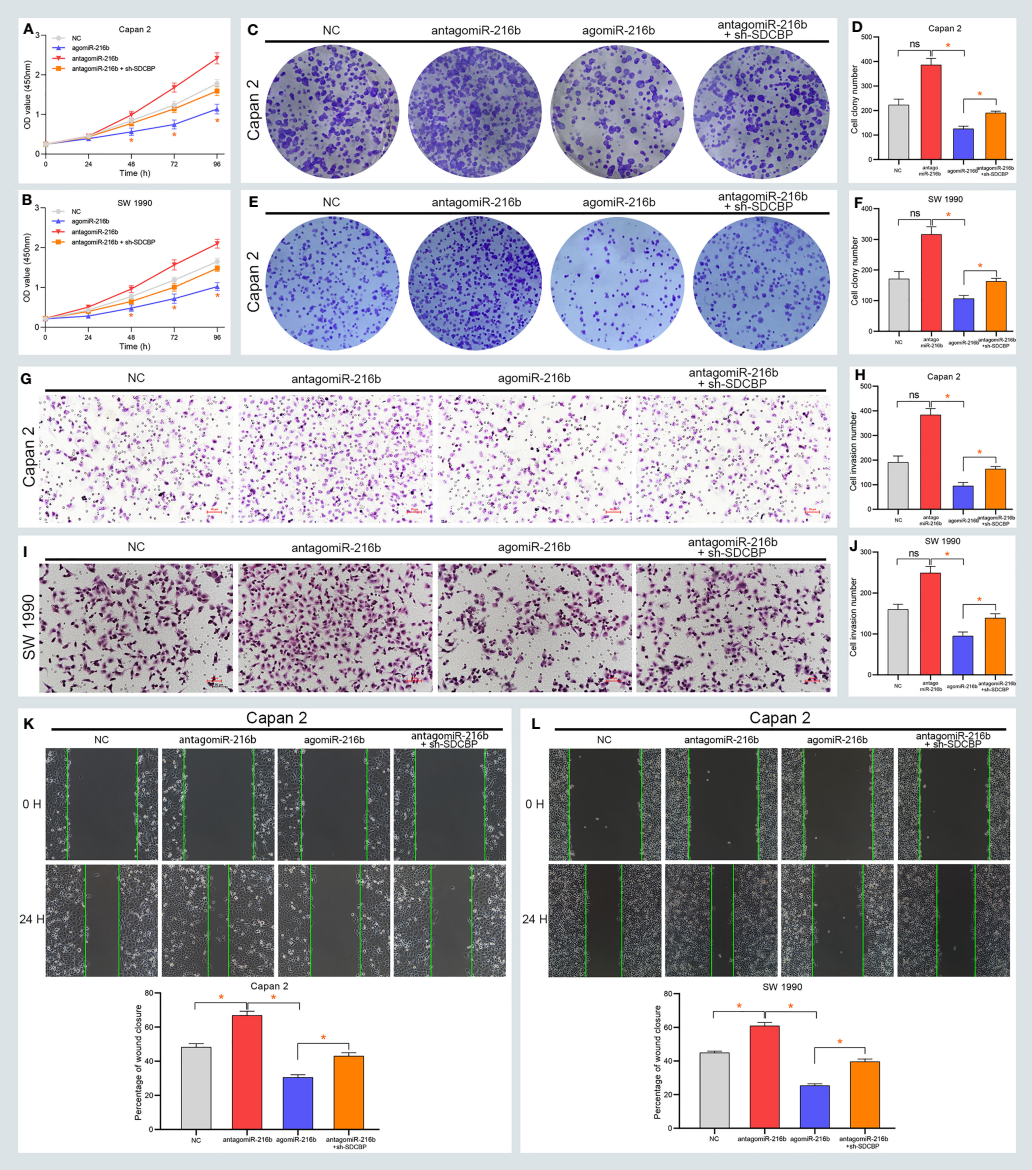
MiR-216b regulates PC progression *via* SDCBP. **(A, B)** The proliferation of PC cells was inhibited and promoted after agomiR-216b and antagomiR-216b transfection, respectively. The promotion of antagomiR-216b in PC proliferation can be rescued through SDCBP knockdown. The upregulation and downregulation of miR-216b played suppressed and promoted roles in PC colony formation **(C–F)**, invasion **(G–J)**, and migration **(K, L)**. The promoted roles of antagomiR-216b can be counteracted through SDCBP knockdown in PC colony formation **(C–F)**, invasion **(G–J)**, and migration **(K, L)**. ns means no statistically significant. *Means P < 0.05.

To further explore the roles of miR-216b in PC, we up- and downregulated its expression using agomiR-216b and antagomiR-216b, respectively. As shown in **Figures 4A, B**, the proliferation of Capan-2 and SW1990 cells was markedly inhibited following transfection with agomiR-216b. However, the proliferation of PC cells was notably increased following transfection with antagomiR-216b. Additionally, the inducing effect of antagomiR-216b on PC proliferation could be rescued through SDCBP knockdown (**Figures 4A, B**). In the colony formation assay, the number of colonies formed was suppressed and increased following transfection with agomiR-216b and antagomiR-216b (**Figures 4C–F**). Colony number in PC cells following transfection with antagomiR-216b was also restored by SDCBP knockdown. Moreover, the migration and invasion of PC cells were inhibited and promoted following transfection with agomiR-216b and antagomiR-216b, respectively, and was recovered following SDCBP knockdown (**Figures 4G–L**). These results indicated that miR-216b regulated PC proliferation, migration, and invasion by directly targeting SDCBP.

### MiR-216b Modulated PC EMT Through the PI3K/AKT Pathway

**Figure 5:**
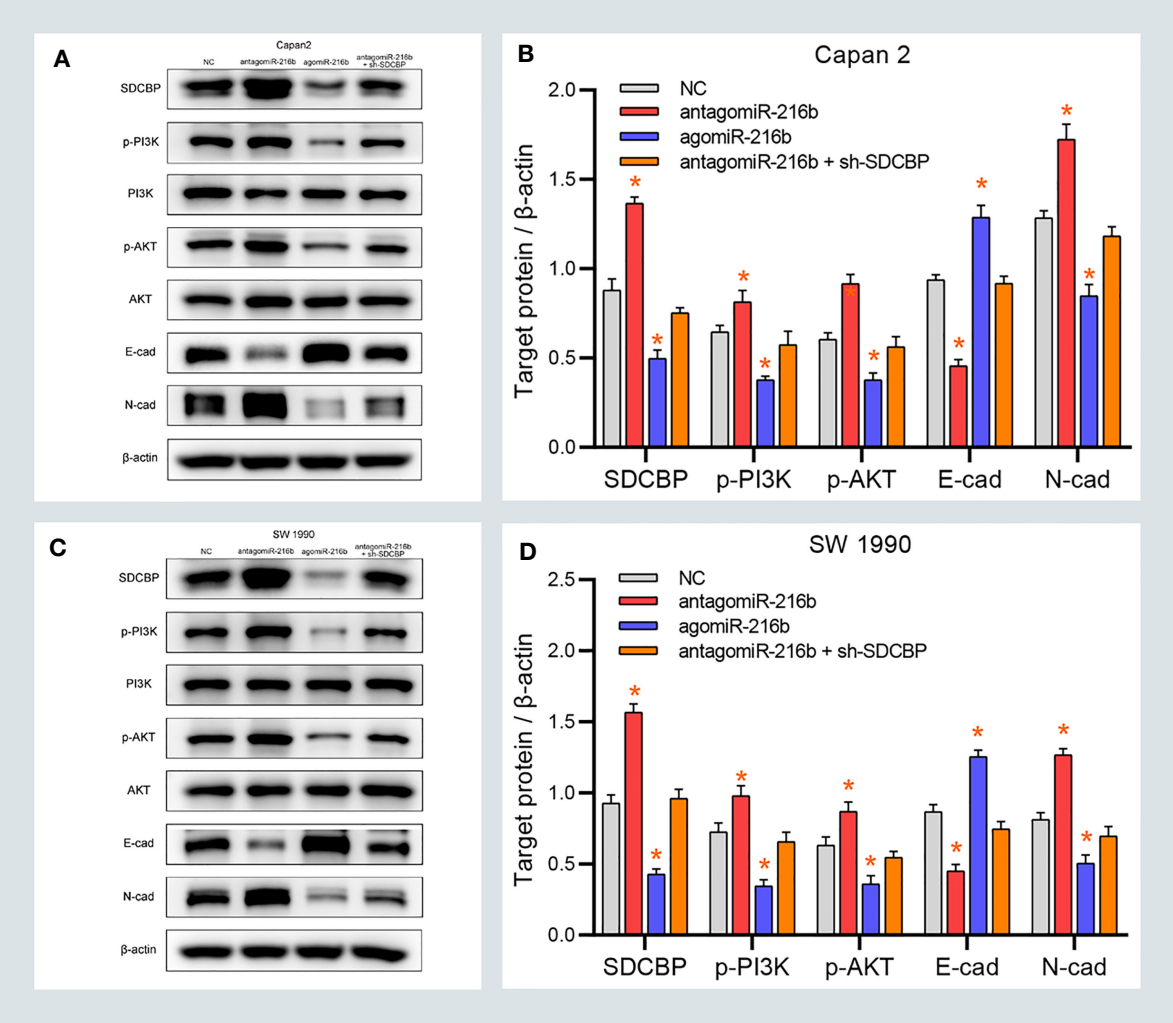
MiR-216b modulates PC EMT through the PI3K/AKT pathway. **(A–D)** The protein levels of p-PI3K, p-AKT, and N-cadherin were upregulated and downregulated after SDCBP antagomiR-216b and agomiR-216b transfection, respectively. However, E-cadherin protein levels were opposite to N-cadherin. There were no changes in PI3K and AKT protein levels. Those changes in antagomiR-216b can be rescued from SDCBP knockdown **(A–D)**. *Means P < 0.05.

The previous results revealed that SDCBP promoted EMT of PC cells *via* the PI3K/AKT pathway. Considering that SDCBP was identified as a direct target of miR-216b, we validated the roles of miR-216b in EMT and the PI3K/AKT pathway. As shown in **Figures 5A–D**, some proteins were notably down- and upregulated following transfection with agomiR-216b and antagomiR-216b, respectively, including p-PI3K, p-AKT, and N-cadherin. Furthermore, those changes in the promotion of miR-216b could be rescued from SDCBP knockdown. However, there were no changes in the expression of PI3K and AKT proteins. Notably, expression of E-cadherin also changed following transfection with agomiR-216b and antagomiR-216b. Together, our findings indicated that targeting miR-216b with SDCBP induced EMT in PC through the PI3K/AKT pathway.

### MiR-216b Inhibited PC Growth *In Vivo*

**Figure 6:**
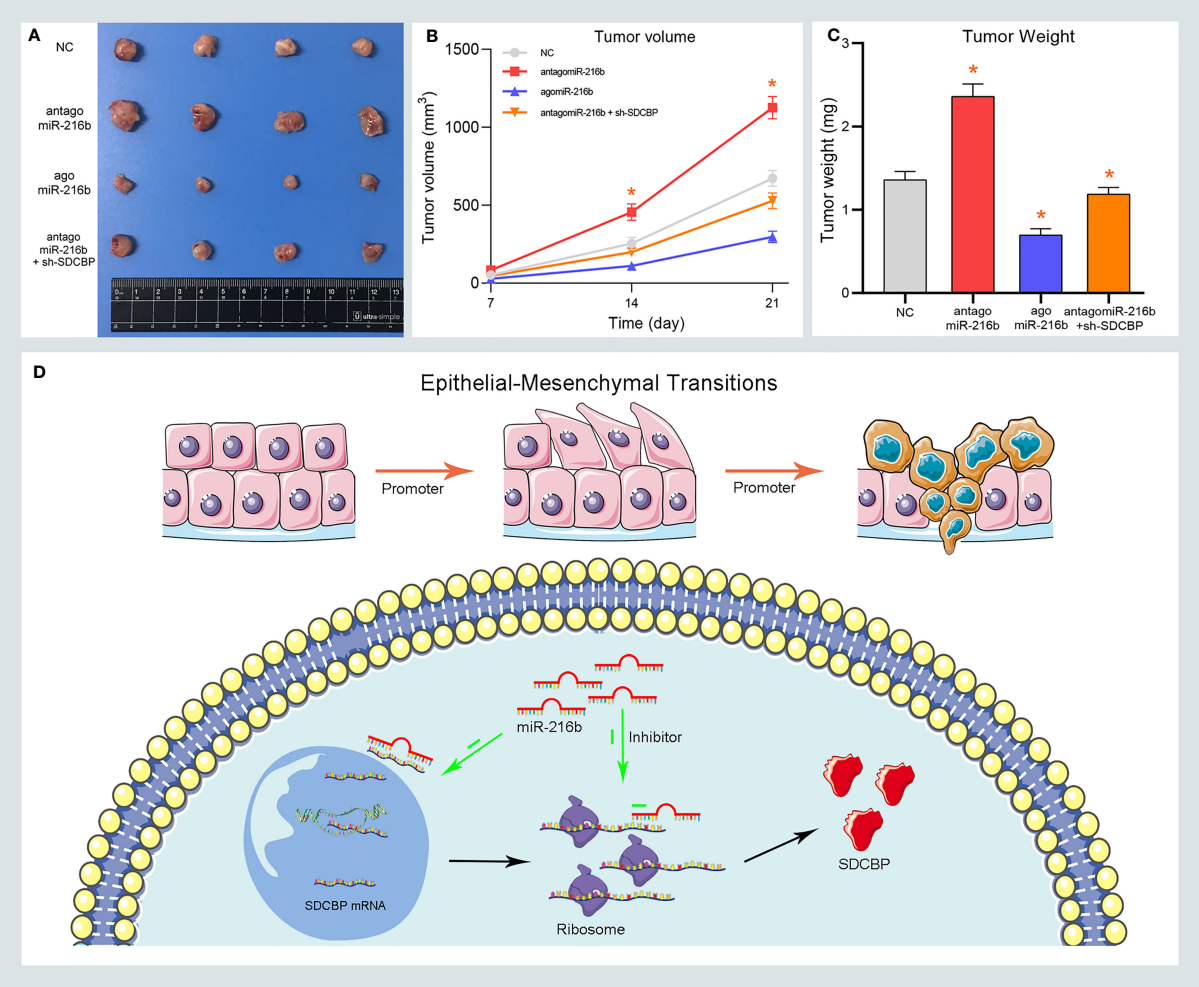
MiR-216b inhibits PC growth by targeting SDCBP *in vivo*. **(A–C)** Tumor volume and weight were promoted and inhibited after antagomiR-216b and agomiR-216b transfection, respectively. The promotion of antagomiR-216b in tumor volume and weight could be counteracted by SDCBP knockdown. **(D)** The schematic representations of miR-216b directly targeting SDCBP to regulate PC progression and EMT. *Means P < 0.05.

To confirm the role of miR-216b *in vivo*, xenograft tumors were established in BALB/c nude mice. Mice were subcutaneously injected with Capan2 cells and then divided into four groups, as follows: NC, antagomiR-216b, agomiR-216b, and antagomiR-216b + sh-SDCBP. We evaluated changes in tumor volume and weight in each group. As shown in **Figures 6A, B**, tumor volume was markedly up- and downregulated following transfection with antagomiR-216b and agomiR-216b, respectively. Furthermore, antagomiR-216b significantly reduced tumor volume following SDCBP knockdown. The inhibitory effect of SDCBP on tumor volume could be counteracted by transfection with antagomiR-216b. This response was also consistent with regard to tumor weight (**Figure 6C**). These findings revealed that miR-216b directly targeted SDCBP to regulate PC progression and EMT. The interaction between SDCBP and miR-216b is presented schematically in **Figure 6D**. SDCBP promotes PC progression by inducing EMT, and was negatively regulated by miR-216b at the post-transcriptional level.

## Discussion

PC is associated with poor survival; therefore, we investigated the potential mechanism underlying the proliferation, migration, and invasion of PC. Previously, we identified 161 DEPs between PC cells with high and low invasive and metastatic potential. To further explore the roles of these DEPs in PC, we validated their expression and evaluated their relationship with prognosis through survival and Cox analyses. We identified proteins associated with survival, namely, CLIC1, KRT7, and SDCBP. Previous studies have shown that both CLIC1 and KRT7 play vital roles in PC progression. For example, CLIC1 overexpression in PC was associated with worse OS (46). Furthermore, *CLIC1* knockdown inhibited the proliferation, migration, and invasion of PC cells (8). KRT7 has been identified as a prognostic factor in PC (47). KRT7 transcription is activated by WT1 expression, which promote the migration and invasion of PC. Furthermore, silencing KRT7 was found to diminish this progression (9). However, the roles of SDCBP in PC remain unclear.

SDCBP is a multifunctional scaffold protein, which promotes the migration and invasion of cancer cells by inducing EMT (11, 12). EMT endows cells with the ability to invade and metastasize, which occurs during the progression and metastasis of PC (16, 48). However, the relationship between SDCBP and EMT remains unclear. As revealed in the GSEA, we predicted that SDCBP might be involved in EMT (**Supplementary Figures S2F, G**). Moreover, the findings of previous studies also support the effect of SDCBP on EMT. For example, SDCBP was found to modulate RhoA and Cdc42 expression *via* TGF-β1, which induced EMT and promoted breast cancer metastasis (49). SDCBP upregulates Slug expression, thereby mediating suppression of E-cadherin to induce EMT in lung cancer (50). SDCBP was found to mediate chemoresistance and stemness in prostate cancer stem cells *via* the activation of STAT3 (51). In the present study, we confirmed that high expression of SDCBP promoted PC proliferation, migration, and invasion, and induced EMT through the PI3K/AKT pathway. These findings were consistent with our prediction and the results of previous studies, which revealed the importance of SDCBP in the proliferation, migration, and invasion of PC, and in the induction of EMT.

MicroRNAs regulate protein expression and play essential roles in the progression of PC (52). To elucidate the relationship between SDCBP and miRNAs, we predicted and validated the upstream miRNA of SDCBP. hsa-miR-216b was found to directly target SDCBP. The low expression of miR-216b promoted the proliferation, migration, and invasion of PC. Luciferase reporter assays revealed that miR-216bis directly targeted by SDCBP. Furthermore, targeting miR-216b with SDCBP induced PC EMT through the PI3K/AKT pathway. Our findings are supported by those of previous studies. For example, miR-216b suppressed PC progression by targeting TPT1 (30). MiR-216b targeted FGFR1 and conferred PC sensitivity to radiotherapy (53). As shown by Egeli et al., miR-216b restrained PC growth, migration, and invasion by directly targeting ROCK1 (54). Thus, targeting miR-216b with SDCBP modulates the proliferation, migration, and invasion of PC, and induces EMT through the PI3K/AKT pathway. Additionally, we identified hsa-miR-139 and hsa-miR-155 as upstream miRNAs of SDCBP, which exert crucial regulatory roles during PC progression. As shown by Zhu et al., miR-139-5p/SLC7A11 inhibited PC proliferation, invasion, and metastasis *via* the PI3K/AKT pathway (55). PC-secreted miR-155 was found to target the TP53INP1 protein in fibroblasts and contribute to fibroblast activation (56). The results of those studies suggested the vital roles of SDCBP in PC progression. Our findings revealed that the miR-216b/SDCBP axis promoted PC progression and induced EMT *via* the PI3K/AKT pathway, representing potential future therapeutic targets.

Our research had some limitations. For example, although we measured the expression of SDCBP in tissue samples, further investigations are needed to determine whether differences exist in PC plasma and metastatic specimens. The prognostic roles of miR-216b were not evaluated due to the limited availability of fresh specimens. To increase the reliability of our findings, we validated expression and prognostic data using TCGA, GTEx, and GEO databases. Our validation was found to be consistent with our prediction. Additionally, we evaluated the roles of miR-216b and SDCBP in the progression of PC using subcutaneous tumors rather than tail vein injection assay. However, we validated the roles of SDCBP in PC progression through SW1990, which was established by PC with spleen metastasis. These findings indicated that SDCBP might be involved in PC metastasis. Further research is needed to elucidate this interaction. Generally, we found that downregulation of the expression of miR-216b and the oncogene SDCBP promotes PC proliferation, migration, invasion, and induces EMT through the PI3K/AKT pathway, which enhances our understanding of miR-216b and SDCBP in PC progression.

## Conclusions

In conclusion, we demonstrated that SDCBP promoted PC migration and invasion, and induced EMT through the PI3K/AKT pathway. SDCBP was identified as a direct target of miR-216b. The downregulation of miR-216b inhibited PC progression and induced EMT through the PI3K/AKT pathway by targeting SDCBP. Finally, SDCBP knockdown counteracted the promotion of antagomiR-216b in PC migration and invasion. These findings indicated that the miR-216b/SDCBP axis contributes to PC proliferation, migration, and invasion, which may represent future therapeutic targets.

## Data Availability Statement

The original contributions presented in the study are included in the article/**Supplementary Material**. Further inquiries can be directed to the corresponding author.

## Ethics Statement

The studies involving human participants were reviewed and approved by the Ethics Committee of Shengjing Hospital of China Medical University. The patients/participants provided their written informed consent to participate in this study. The animal study was reviewed and approved by Ethics Committee of Shengjing Hospital of China Medical University.

## Author Contributions

FZ, QL, HC, and XT conceived and designed the experiments. FZ, HC, HZ, and ZL performed the data analysis. FZ, HC, and QL prepared the manuscript. XT critically read and discussed the manuscript. All authors have read and agreed to the published version of the manuscript.

## Funding

This work was supported by the National Natural Science Foundation of China (No. 180530068), the Special Project of Provincial Science and Technology Development from National Guidance (No. 2020JH6/10500055), the Key Research and Development Program of Liaoning province (2020JH2/10300130), the Key Research and Development Program of Liaoning province (2021JH2/10300010), and the Outstanding Doctor Foundation of China Medical University (M0554).

## Conflict of Interest

The authors declare that the research was conducted in the absence of any commercial or financial relationships that could be construed as a potential conflict of interest.

## Publisher’s Note

All claims expressed in this article are solely those of the authors and do not necessarily represent those of their affiliated organizations, or those of the publisher, the editors and the reviewers. Any product that may be evaluated in this article, or claim that may be made by its manufacturer, is not guaranteed or endorsed by the publisher.

## Supplementary Material

Supplementary Figure S1 Identifying differentially expressed proteins. **(A–C)** The volcano plots of DEGs in the TCGA_GTEx group, GSE62165 group, and GSE15471 group with thresholds of |log2FC| > 1, and adjust P-value < 0.05, respectively. The red dots and blue dots separately represent the up-regulated and down-regulated DEGs. **(D)** The intersection of DEGs in three groups and 161 DEPs.

Supplementary Figure S2 Screening survival-related proteins and functional analysis. **(A, B)** Identification of three survival-related proteins through univariate and multivariate cox analysis, respectively. **(C–E)** Validating the expression roles and prognosis values of three survival-related proteins including CLIC1 **(C)**, SDCBP **(D)** and KRT7 **(E)**. **(F, G)** Functional analysis of SDCBP in Gene Ontology **(F)** and KEGG pathway **(G)**.

Supplementary Figure S3 Predicting and assessing the upstream miRNA of SDCBP. **(A)** The volcano plots of DEMIs in the TCGA group with thresholds of |log2FC| > 1, and adjust P-value < 0.05. **(B)** The common miRNAs in the prediction of TargetScan, miRNet and DEMIs. **(C, D)** Validating the expression roles and prognosis values of miR-216b. **(E)** Correlation analysis indicated SDCBP was negatively correlated with miR-216b.
