# Supplementary figures and images for: Syntenin Regulated by miR-216b Promotes Cancer Progression in Pancreatic Cancer
Source: Front Oncol. 2022 Jan 28;12:790788. doi: 10.3389/fonc.2022.790788 (PMC8831246; doi:10.3389/fonc.2022.790788)

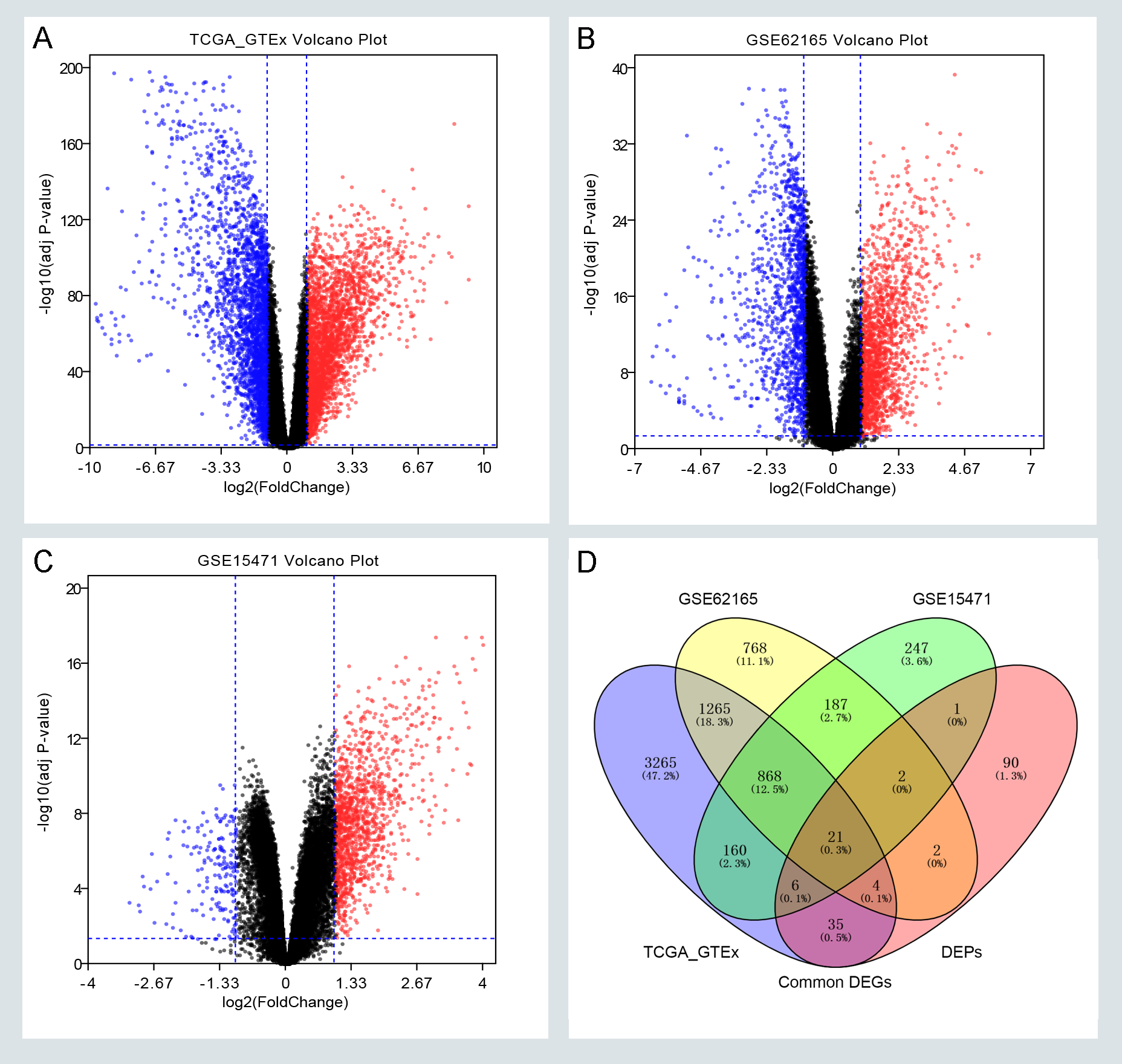

Supplement: Supplementary Figure S1 — Identifying differentially expressed proteins. (A–C) The volcano plots of DEGs in the TCGA_GTEx group, GSE62165 group, and GSE15471 group with thresholds of |log2FC| > 1, and adjust P-value < 0.05, respectively. The red dots and blue dots separately represent the up-regulated and down-regulated DEGs. (D) The intersection of DEGs in three groups and 161 DEPs. [file Image_1.tif]

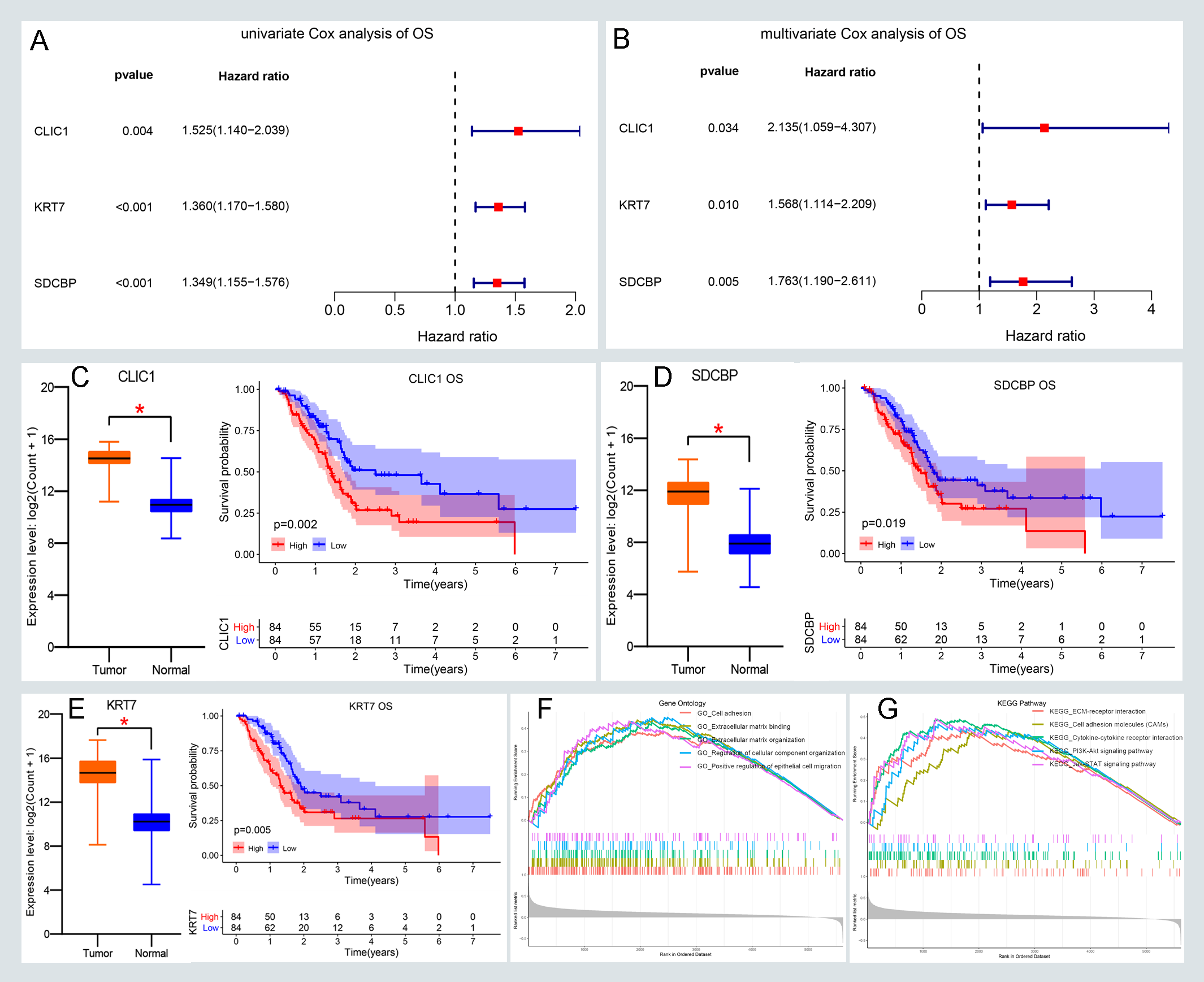

Supplement: Supplementary Figure S2 — Screening survival-related proteins and functional analysis. (A, B) Identification of three survival-related proteins through univariate and multivariate cox analysis, respectively. (C–E) Validating the expression roles and prognosis values of three survival-related proteins including CLIC1 (C), SDCBP (D) and KRT7 (E). (F, G) Functional analysis of SDCBP in Gene Ontology (F) and KEGG pathway (G). [file Image_2.tif]

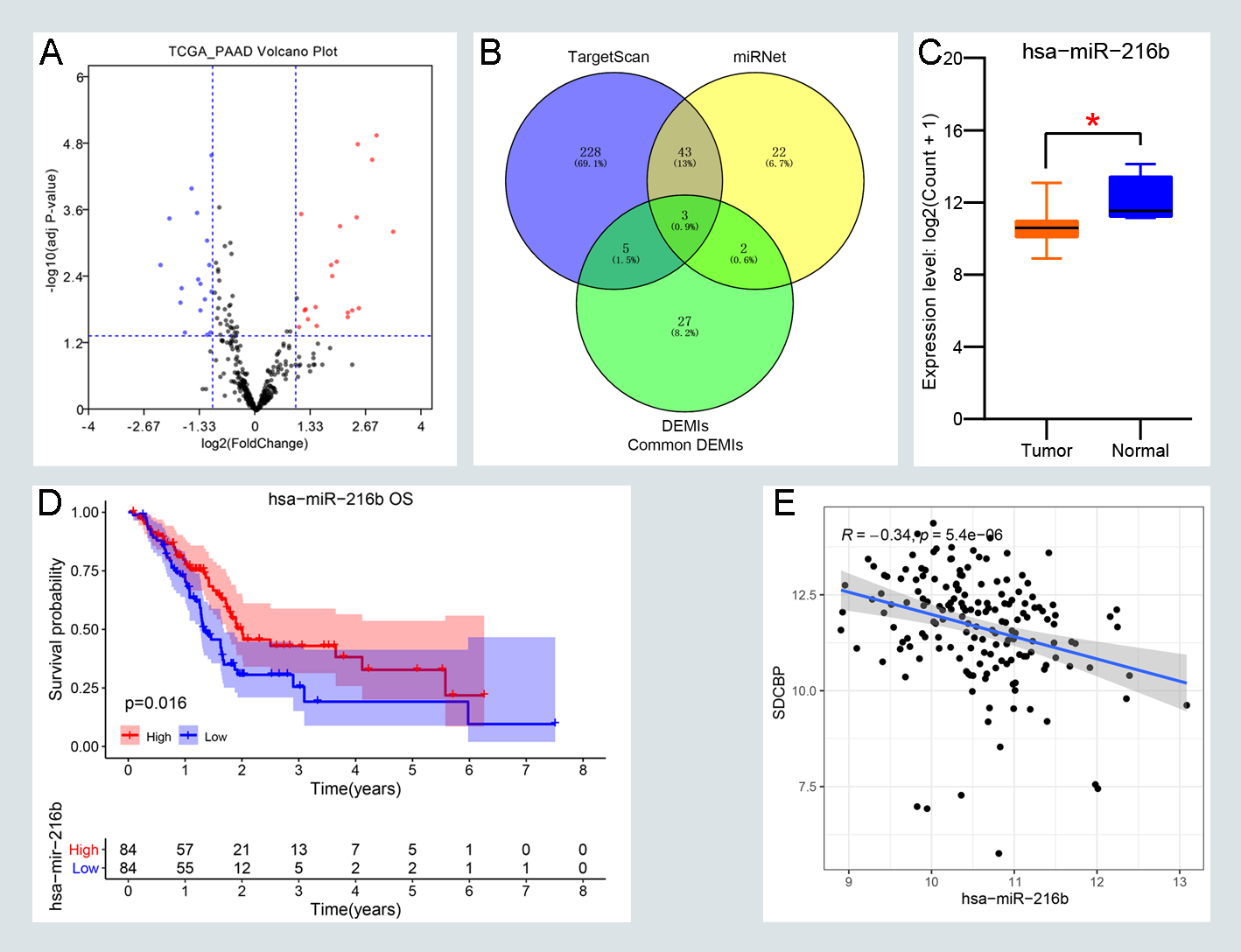

Supplement: Supplementary Figure S3 — Predicting and assessing the upstream miRNA of SDCBP. (A) The volcano plots of DEMIs in the TCGA group with thresholds of |log2FC| > 1, and adjust P-value < 0.05. (B) The common miRNAs in the prediction of TargetScan, miRNet and DEMIs. (C, D) Validating the expression roles and prognosis values of miR-216b. (E) Correlation analysis indicated SDCBP was negatively correlated with miR-216b. [file Image_3.tif]
