# Supplementary material for: Syntenin Regulated by miR-216b Promotes Cancer Progression in Pancreatic Cancer
Source: Front Oncol. 2022 Jan 28;12:790788. doi: 10.3389/fonc.2022.790788 (PMC8831246; doi:10.3389/fonc.2022.790788)
Supplement: Supplementary file 4 [file DataSheet_1.pdf]

Supplementary Data： Whole Western blot images

Syntenin regulated by miR-216b promotes cancer progression in pancreatic cancer

Fuqiang Zu [1#], Qingfeng Liu [2#], Hui Chen [1], Hui Zang [2], Zeyu Li [1], Xiaodong Tan [1*]

Figure 1 J

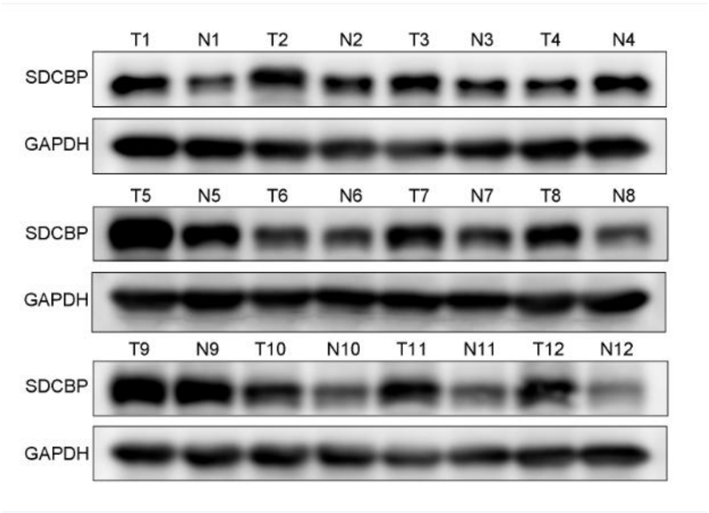

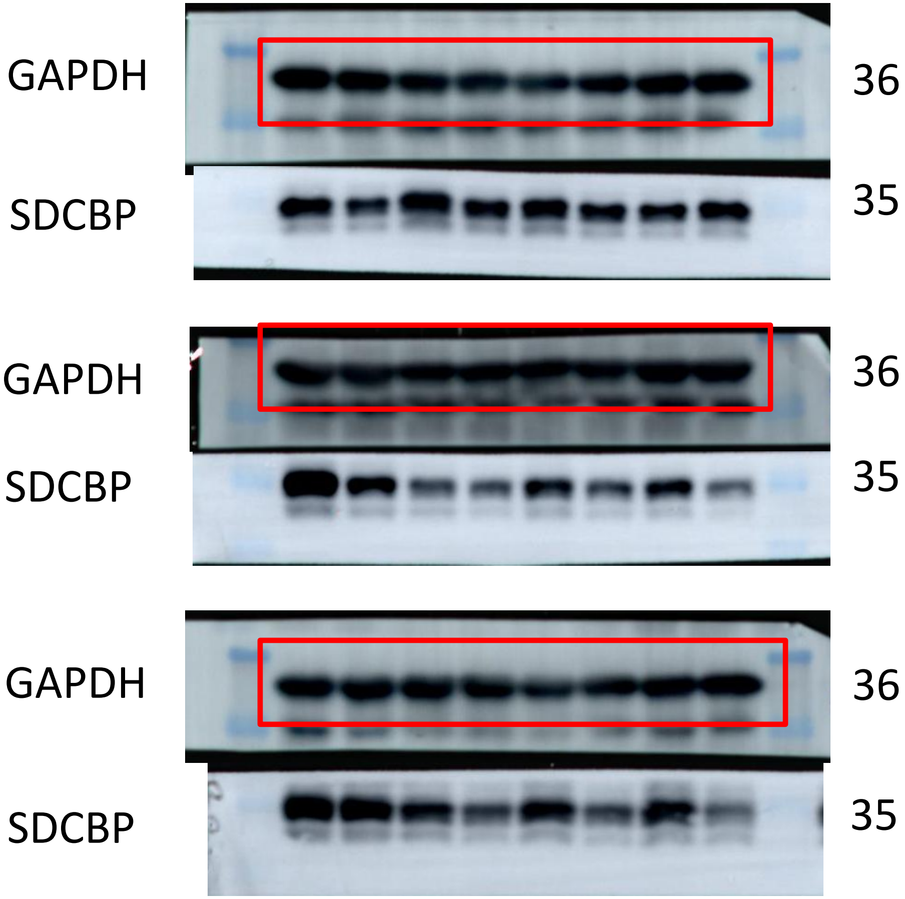

Figure 2A

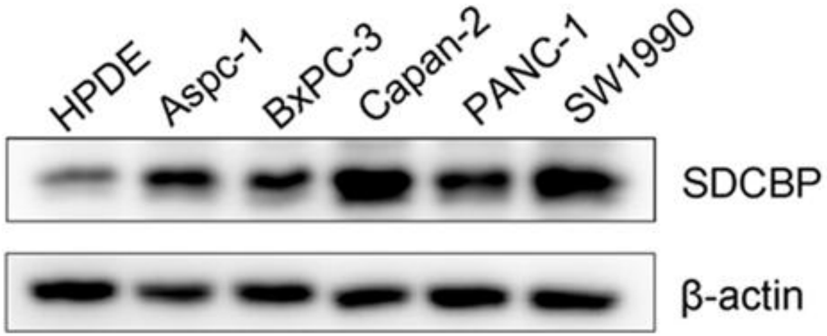
HPDE
Aspc-1
BxPC-3
Capan-2
PANC-1
SW1990
SDCBP
β-actin

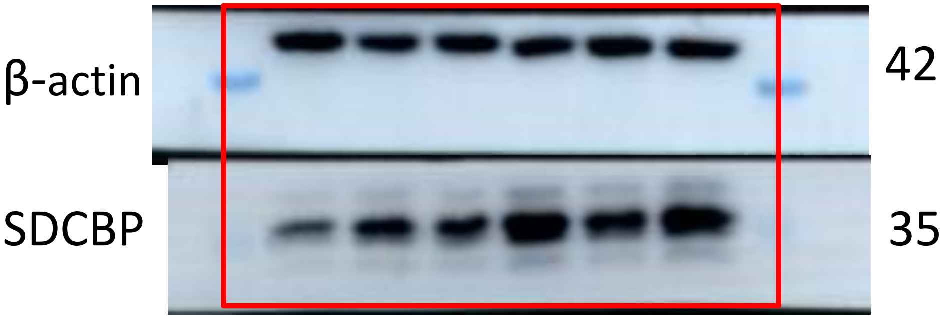
β-actin
SDCBP
42
35

Figure 3A

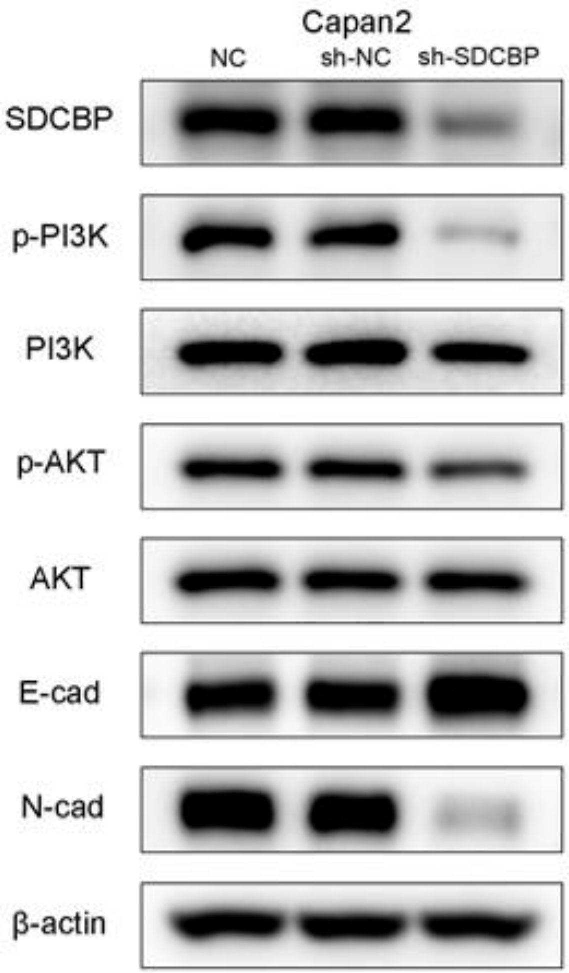

Capan2
NC
sh-NC
sh-SDCBP
SDCBP
p-PI3K
PI3K
p-AKT
AKT
E-cad
N-cad
β-actin

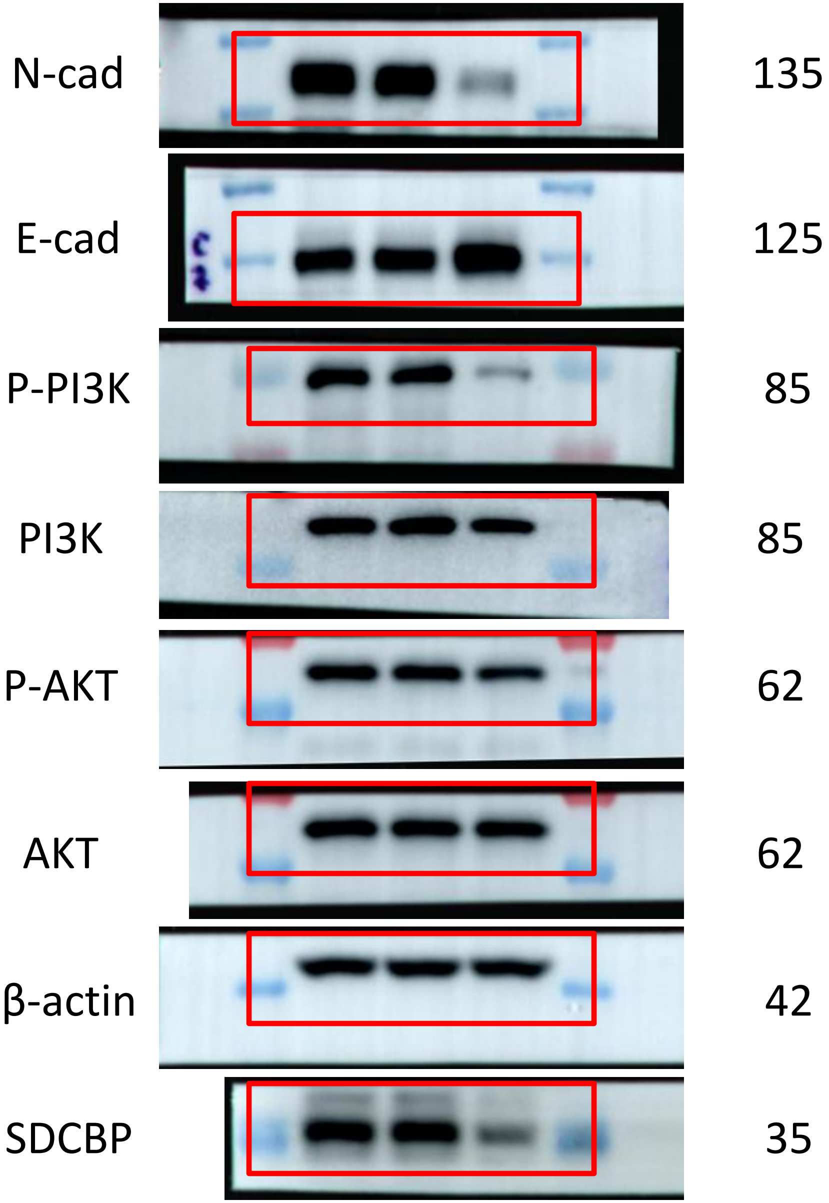

N-cad
135
E-cad
125
P-PI3K
85
PI3K
85
P-AKT
62
AKT
62
β-actin
42
SDCBP
35

Figure 3C

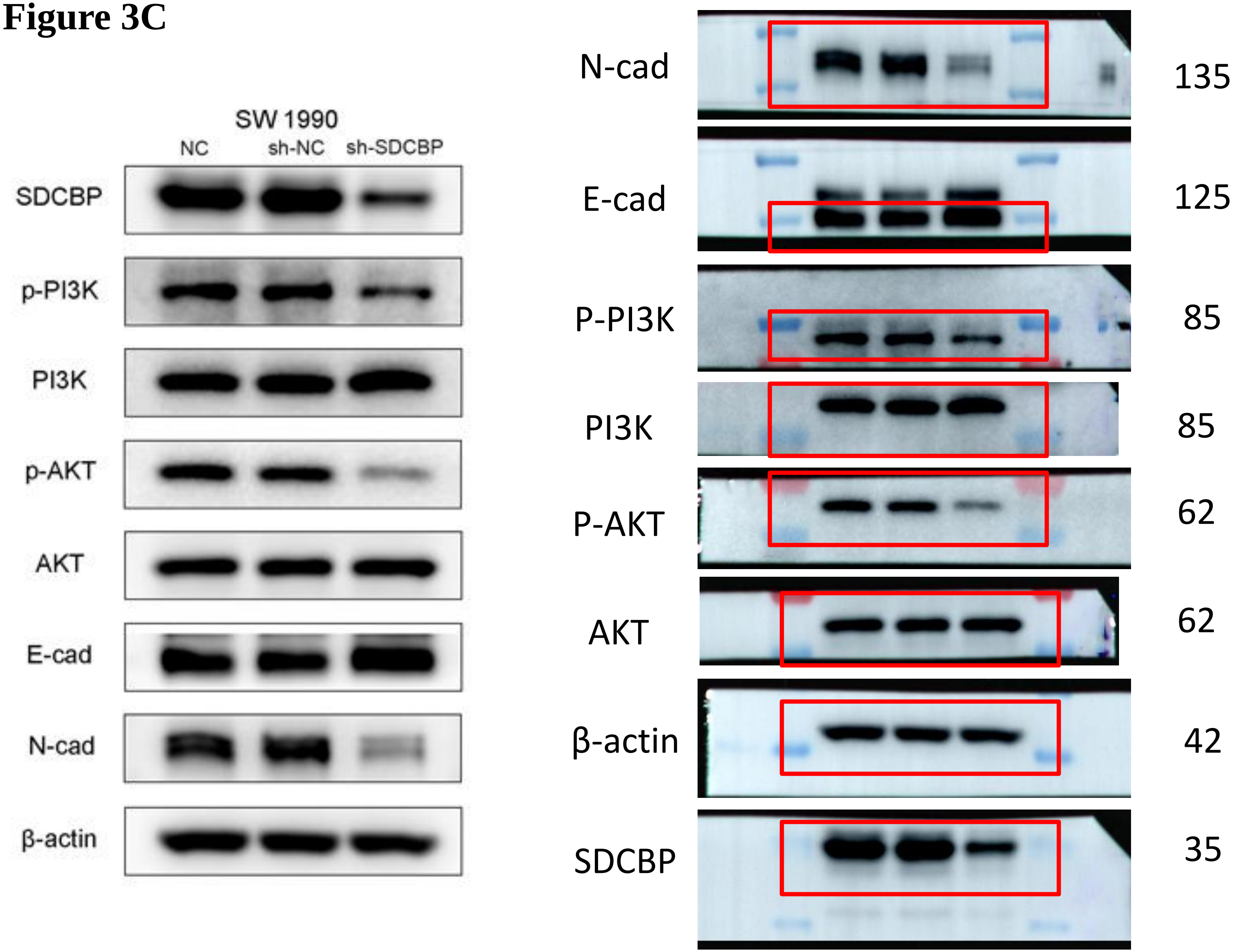
SW 1990
NC
sh-NC
sh-SDCBP
SDCBP
p-PI3K
PI3K
p-AKT
AKT
E-cad
N-cad
β-actin
N-cad
135
E-cad
125
P-PI3K
85
PI3K
85
P-AKT
62
AKT
62
β-actin
42
SDCBP
35

Figure 5A

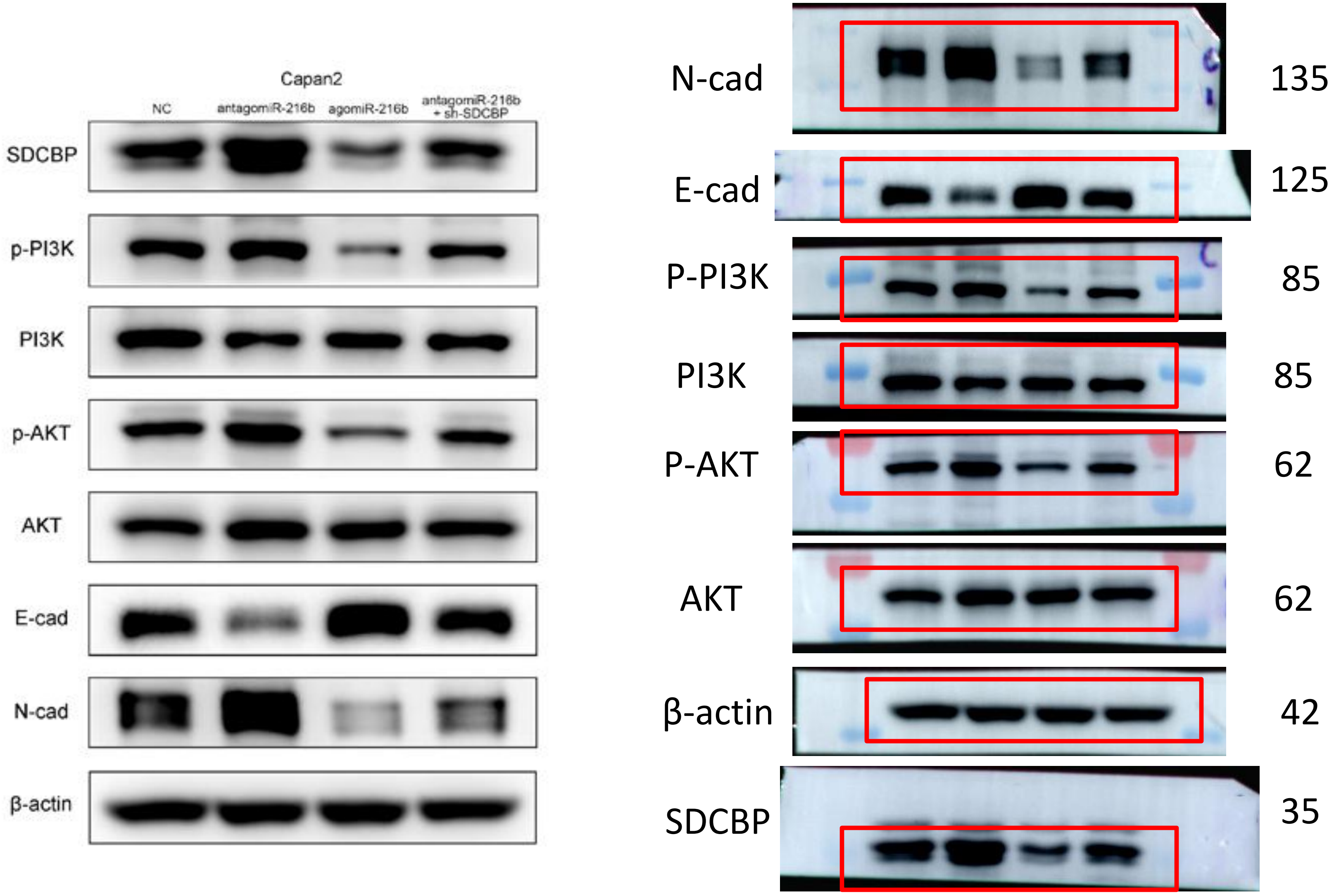
Capan2
NC
antagomiR-216b
agomiR-216b
antagomiR-216b + sh-SDCBP
SDCBP
p-PI3K
PI3K
p-AKT
AKT
E-cad
N-cad
β-actin
N-cad
135
E-cad
125
P-PI3K
85
PI3K
85
P-AKT
62
AKT
62
β-actin
42
SDCBP
35

Figure 5C

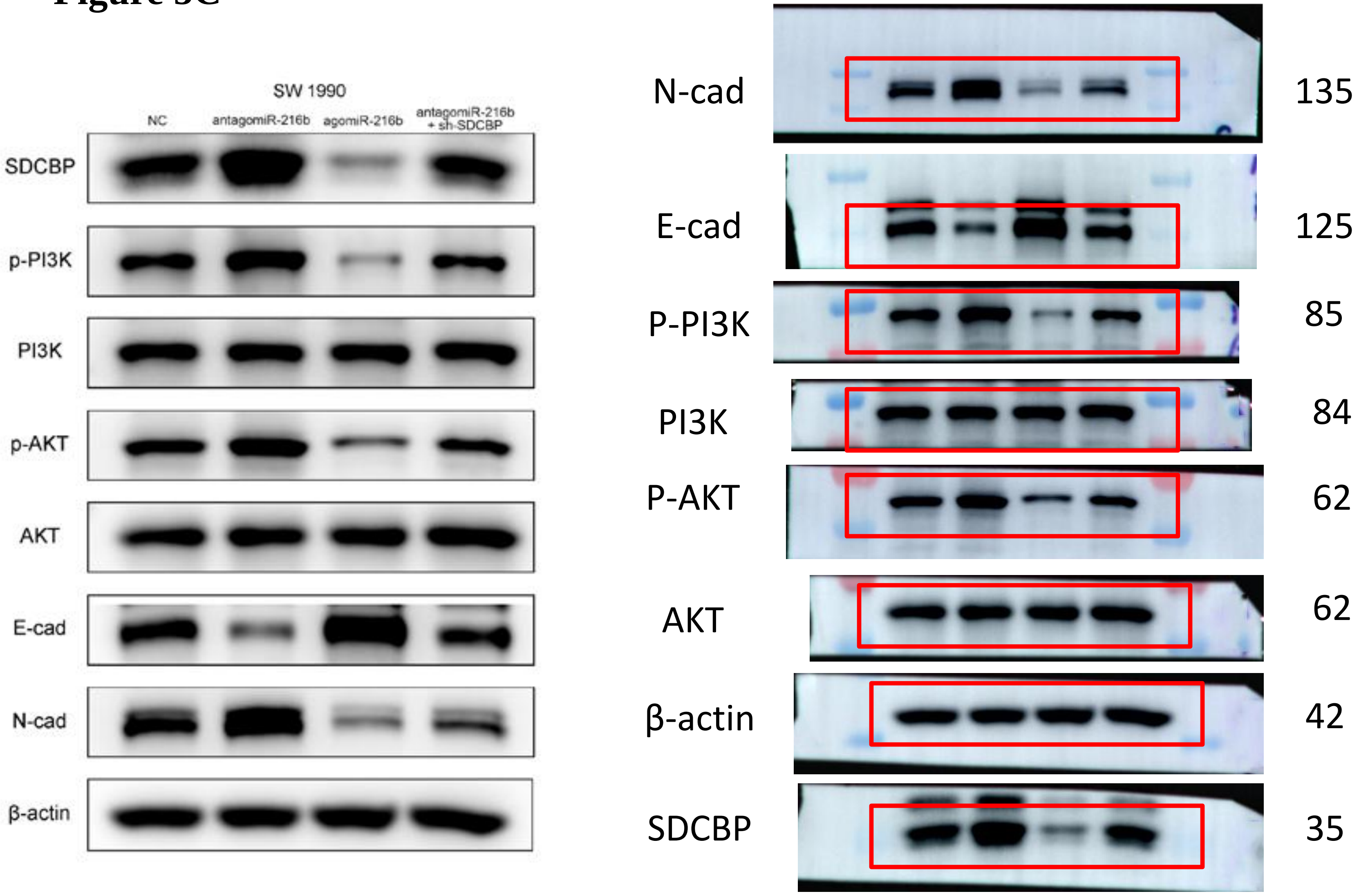
SW 1990
NC
antagomiR-216b
agomiR-216b
antagomiR-216b + sh-SDCBP
SDCBP
p-PI3K
PI3K
p-AKT
AKT
E-cad
N-cad
β-actin
N-cad
135
E-cad
125
P-PI3K
85
PI3K
84
P-AKT
62
AKT
62
β-actin
42
SDCBP
35
