# Supplementary material for: Syntenin Regulated by miR-216b Promotes Cancer Progression in Pancreatic Cancer
Source: Front Oncol. 2022 Jan 28;12:790788. doi: 10.3389/fonc.2022.790788 (PMC8831246; doi:10.3389/fonc.2022.790788)
Supplement: Supplementary file 6 [file Table_2.docx]

Table S2: Details of GEO, TCGA, and GTEx databases are included in this study.

| Platform | Datasets | Sample size | | | Differentially expressed genes | | |
| --- | --- | --- | --- | --- | --- | --- | --- |
|  |  | Total | Tumor | Normal | Total | UP-DEGs | Down-DEGs |
| GPL570 | GSE15471 | 72 | 36 | 36 | 1492 | 1307 | 185 |
| GPL13667 | GSE62165 | 131 | 118 | 13 | 3117 | 1712 | 1405 |
| TCGA_GTEx | TCGA | 182 | 178 | 4 | 5624 | 2863 | 2761 |
|  | GTEx | 167 | 0 | 167 |  |  |  |

DEGs: Differentially expressed genes with thresholds of |log2FC| >1 and adjust P value < 0.05
