# Supplementary material for: Syntenin Regulated by miR-216b Promotes Cancer Progression in Pancreatic Cancer
Source: Front Oncol. 2022 Jan 28;12:790788. doi: 10.3389/fonc.2022.790788 (PMC8831246; doi:10.3389/fonc.2022.790788)
Supplement: Supplementary file 7 [file Table_3.docx]

Table S3: Clinical characteristics of our study.

| **Characteristics** | No. of patients | SDCBP | | P value |
| --- | --- | --- | --- | --- |
|  |  | Low | High |  |
| **Cases** | 61 | 20 | 41 |  |
| Age (years) |  |  |  | 0.746 |
| ≤ 60 | 20 | 6 | 14 |  |
| > 60 | 41 | 14 | 27 |  |
| **Gender** |  |  |  | 0.793 |
| Female | 26 | 9 | 17 |  |
| Male | 35 | 11 | 24 |  |
| **Tumor location** |  |  |  | 0.400 |
| Head | 48 | 17 | 31 |  |
| Body and tail | 13 | 3 | 10 |  |
| **Histologic grade** |  |  |  | 0.746 |
| G 1 | 41 | 14 | 27 |  |
| G 2 + G 3 | 20 | 6 | 14 |  |
| **Tumor stage** |  |  |  | 0.465 |
| T1 + T2 | 12 | 5 | 7 |  |
| T3 | 49 | 15 | 34 |  |
| **Lymph stage** |  |  |  | 0.0640 |
| N0 (negative) | 18 | 9 | 9 |  |
| N1 (positive) | 43 | 11 | 32 |  |
| **AJCC stage** |  |  |  | 0.0640 |
| Stage I + IIA | 18 | 9 | 9 |  |
| Stage IIB + III | 43 | 11 | 32 |  |
| **Survival outcome** |  |  |  | 0.921 |
| Alive | 28 | 9 | 19 |  |
| Death | 33 | 11 | 22 |  |

Abbreviations: Syntenin, SDCBP; PC, pancreatic cancer; AJCC, American Joint Committee on Cancer. G 1, high differentiation; G 2, moderate differentiation; G 3, low differentiation.
